# Improving the biopharmaceutical attributes of mangiferin using vitamin E-TPGS co-loaded self-assembled phosholipidic nano-mixed micellar systems

**DOI:** 10.1007/s13346-018-0498-4

**Published:** 2018-04-10

**Authors:** Rajneet Kaur Khurana, Balan Louis Gaspar, Gail Welsby, O. P. Katare, Kamalinder K. Singh, Bhupinder Singh

**Affiliations:** 1 University Institute of Pharmaceutical Sciences, Panjab University, Chandigarh, 160014 India; 2 Department of Histopathology, Post Graduate Institute of Medical Education and Research (PGIMER), Chandigarh, 160012 India; 3 School of Pharmacy and Biomedical Sciences, Faculty of Clinical and Biomedical Sciences, University of Central Lancashire, Preston, PR1 2HE UK; 4 UGC-Centre of Excellence in Applications of Nanomaterials, Nanoparticles and Nanocomposites (Biomedical Sciences), Panjab University, Chandigarh, 160014 India

**Keywords:** Breast cancer, Quality by design (QbD), Mangiferin, Vitamin E TPGS nanomicelles, Self-assembled phospholipidic nano-mixed miceller system (SPNMS), Pharmacokinetics, Bioavailability, P-gp efflux, Cellular uptake

## Abstract

The current research work encompasses the development, characterization, and evaluation of self-assembled phospholipidic nano-mixed miceller system (SPNMS) of a poorly soluble BCS Class IV xanthone bioactive, mangiferin (Mgf) functionalized with co-delivery of vitamin E TPGS. Systematic optimization using I-optimal design yielded self-assembled phospholipidic nano-micelles with a particle size of < 60 nm and > 80% of drug release in 15 min. The cytotoxicity and cellular uptake studies performed using MCF-7 and MDA-MB-231 cell lines demonstrated greater kill and faster cellular uptake. The ex vivo intestinal permeability revealed higher lymphatic uptake, while in situ perfusion and in vivo pharmacokinetic studies indicated nearly 6.6- and 3.0-folds augmentation in permeability and bioavailability of Mgf. In a nutshell, vitamin E functionalized SPNMS of Mgf improved the biopharmaceutical performance of Mgf in rats for enhanced anticancer potency.

**Electronic supplementary material:**

The online version of this article (10.1007/s13346-018-0498-4) contains supplementary material, which is available to authorized users.

## Introduction

Mangiferin (Mgf), a naturally produced polyphenol molecule possessing four hydroxyl groups, is an efficient antioxidant for free radical chain termination [1]. Mgf shows potential cytotoxicity effects on cancer cells and may even induce apoptosis by inhibiting and suppressing nuclear factor kappa B (NF-kB) and NF-κB-inducing kinase [2, 3]. Several literature [3, 4] also report Mgf-induced apoptosis, and tumorigenesis through altered gene expression [5–7], especially using Bcl-2 and Bax. Definitive activity of this bioactive phytochemical has also been documented on HL-60 cells programmed cell death, ascribed to suppression of Bcl-xL and XIAP expression and inhibition of the NF-kB pathway [8].

Despite being a very potent antioxidant molecule, Mgf exhibits very low and variable bioavailability (i.e., 1.5 to 5%), owing principally to limited aqueous oral solubility (i.e., 0.1 to 0.3 mg/mL) and poor lipophilicity (i.e., log P of − 0.56), extensive P-gp efflux, high first-pass effect, and considerable metabolism by gut Cytochrome P-450 enzymes [9–14]. By virtue of its low aqueous solubility and lipophilicity, Mgf can be safely regarded as a BCS class IV agent.

Owing to the aforementioned challenges [15], several scientists have attempted to enhance the oral bioavailability of Mgf by formulating its solid dispersions [16], β-cyclodextrin complexes [17, 18], phospholipid complexes [19], and spray-dried encapsulation [5]. None of the conventional formulations, therefore, is considered to be highly satisfactory in completely surmounting the multifactorial issues of solubility, lipophilicity, and eventually the bioavailability of Mgf. However, the increase of Mfg bioavailability compared to the plain drug has previously been observed with nanostructured lipidic carriers (NLCs) carrying Mfg and Mfg-phospholipid complex [20], indicating safety towards Caco-2 cells with enhanced intestinal permeation parameters. Xiao and associates elucidated the augmentation in in vitro antitumor activity of Mfg conferred by Mfg loaded magnetic polymeric microspheres [21].

The phospholipid systems have lately been explored for enhancing the biopharmaceutical performance and therapeutic efficacy of several bioactives exhibiting poor hydrophilicity and lipophilicity [20]. As phospholipids constitute major part of the bio-membrane, these hold good biocompatibility, while acting as a carrier for delivering drugs across the biological barriers. The amphiphilic nature of phospholipids is documented to enhance solubility and permeability of the drugs, thus improving their oral biopharmaceutical performance [22]. Other stellar merits of phospholipidic formulations include ease of preparation, along with high drug loading capacity and long-term stability. Further, the employed emulgents and co-emulgents lead to complete micellization of the system and thus solubilizing the BCS class IV bioactive, leading eventually to improved absorption potential and thus better bioavailability.

Self-assembled Phospholipidic Nano Mixed Miceller System (SPNMS), in this regard, hold tremendous market potential due to ease in their manufacturing, higher cost-effectiveness, improved efficacy, and higher scalability [23]. This is the first report of its own kind that offers an insight to the use of SPNMS for improving the in vitro breast cancer cytotoxicity and augmenting the biopharmaceutical attributes of Mgf.

Lately, SPNMS have demonstrated considerable potential to enhance the oral bioavailability of anticancer bioactives owing to their ability to circumvent the major hiccups faced by the latter [24–26]. Composed of lipidic constituents, water-insoluble (with HLB < 12; used in 0–20 parts) and water-soluble (with HLB > 12; used in 30–80 parts) emulgents, coemulgents, and cosolvents, these systems are known to produce ultrafine micelles (i.e., < 50 nm in size) in the gastrointestinal (GI) fluids [27]. Hence, SPNMS have been considered as one of the most promising technologies for a vast diversity of drugs [28]. The key feature of such systems is their ability to incorporate drugs exhibiting values of low partition coefficient [29, 30]. Vitamin E D-α-tocopheryl polyethylene glycol 1000 succinate (TPGS) has been widely used as an emulsifier in nanoparticle formulation of anticancer drugs leading to high drug encapsulation and substantially high cellular uptake by cancer [31]. It also has a dual role, one as a bioactive and the other as a P-gp inhibitor that can block the cancer cell action of pumping drugs outside of cells and can enhance the anticancer effect [32, 33].

Mixture designs are highly recommended in a delivery system with multiple excipients, wherein the characteristics of the finished product usually depend on the proportion of each substance present, but not on each quantity. Also, optimal designs are preferred when the nature of factor-response relationship is either unknown or is obscure [34]. D-optimal designs, based on the principle of minimization of variance and covariance of parameters, require correct model(s) to be postulated, variable space to be defined, and number of design points to be so chosen that determines the model coefficients with maximum possible efficiency [35].

Attempts were, therefore, made in the current studies for improving the drug loading potential, enhancing dissolution rate, and augmenting the oral bioavailability of Mgf employing systematically prepared functional SPNMS with co-delivery of TPGS, and evaluating these extensively for their biopharmaceutical attributes. Further, its anticancer potential and cellular uptake in mammary adenocarcinoma cell lines (MDA-MB-231 and MCF-7) have also been investigated. Also, toxicity of the developed and blank formulations was evaluated by excising out all the vital organs followed by their histopathological examination. The present research article offers an insight to use the functionalized SPNMS for improving the biopharmaceutical attributes of Mgf. In vitro, in situ, and in vivo studies carried out on the formulations proved these nanostructures to be highly superior to the pure bioactive.

## Materials and methods

Mgf was obtained from M/s International Association on Mangiferin Research (IAMR), Nanning, Guangxi, China. Vitamin E TPGS and poly(ethylene glycol) 200 (PEG 200) were purchased from M/s Sigma-Aldrich, Chemicals Pvt. Ltd., Mumbai, India. Phospholipid 90G was provided as *ex-gratis* from Lipoid GmbH, Germany. Empty gelatin capsule shells (size 00) were supplied as gift samples by M/s ACG Associated Capsules Pvt. Ltd., Mumbai.

### Identifying quality target product profile and critical quality attributes

As the first and foremost step, quality target product profile (QTPP) was embarked upon as the formulation objectives (Supplementary Table 1) to achieve the maximal therapeutic efficacy for enhancing the bioavailability of Mgf. Valid justification(s) were documented for choosing the apt critical quality attributes (CQAs) to subsequently formulate SPNMS of Mgf (Supplementary Table 2), using the systematic approach of Quality by Design (QbD).

### Risk assessment studies

Risk assessment studies were performed to identify, analyze, and assess the possible interaction(s) among Mgf, excipients, and other process parameters. Ishikawa fishbone cause-and-affect diagram was constructed and failure mode effect analysis (FMEA) was carried out in order to earmark the critical material attributes (CMAs) and/or critical process parameters (CPPs) for SPNMS of Mgf [36].

### Equilibrium solubility studies

Solubility studies for Mgf were carried out for 72 h at 37 ± 1 °C in a water bath by adding an excess amount of drug in various water-soluble emulgents, viz. Acconon CC-6, Cremophor RH40, Cremophor EL, Vitamin E TPGS, Labrafil M1944, Labrasol, Tween 80 and Tween 40. Cosolvents employed included Transcutol HP, PEG 200, and propylene glycol. Aliquots of the filtrate were suitably diluted with methanol and analyzed using the HPTLC method previously reported by the authors at a λ_max_ of 262 nm [37].

### Construction of pseudo-ternary phase diagrams

Phase titration studies were conducted on Phospholipid 90G, Vitamin E TPGS (emulgents), and PEG 200 (cosolvent) as the surfactant mixture (Smix; in the ratio 1:0,1:1,2:1, and 3:1) in the varying ratios, ranging from 1 to 9, were titrated with water at 37 °C to attain the maximal emulsification region [38]. Pseudo-ternary diagrams were constructed using the PCP Disso software ver 3.0 (M/s Pune College of Pharmacy, Pune, India).

### Preparation of Mgf SPNMS

SPNMS of Mgf (dose equivalent to 30 mg) were prepared by employing the self-assembly method [39]. Mgf was complexed with Phospholipid 90G (1:1) as per the already developed method [20]. Subsequently, addition of vitamin E TPGS and PEG 200 in ethanolic mixture was carried out using magnetic stirrer at room temperature. Afterwards, butylated hydroxyl toluene (BHT) 0.2% *w*/*w* of the total lipid was dissolved in the above alcoholic solution. The aqueous phase containing phosphate buffer saline (pH 7.4), sodium metabisulphite (0.5% *w*/*w*), and 0.01% of Triton X-100 was poured into the organic phase in a streamlined manner with continuous stirring at 2000 rpm. The dispersion was stirred for 5–10 min after complete addition of the aqueous phase [40, 41].

### QbD-based formulation optimization studies

For systematic optimization of Mgf-SPNMS formulations, an I-optimal mixture design matrix was constructed with 16 runs including five replicates, using the Design Expert® software version 9.0.1 (M/s Stat-Ease, Minneapolis, USA) in order to optimize the amounts of CMAs viz. Phospholipid 90G (X1), vitamin E TPGS (X2), and PEG 200 (X3). Various levels of CMAs were deduced from the pseudoternary phase diagrams. Supplementary Table 3 displays the design matrix for all the prepared formulations containing 30 mg of Mgf. All the prepared formulations were evaluated for Q15, %DE, *D*_nm_, and *T*_emul_ as the CQAs**.**

#### QbD-enabled data analysis and validation

In order to correlate CMAs with CQAs, multiple linear regression analysis (MLRA) was applied to get the coefficients of polynomial equations employing [35]. Various model parameters like *p* value, coefficient of correlation (*R*), and predicted error sum of squares (PRESS) were analyzed. Besides, 3D-response surface and 2-D contour plots were generated to relate CQAs with CMAs. Further, optimum solution was located by numerical optimization using maximization of the desirability function value, close to unity.

### Characterization of SPNMS

#### Self-emulsification time (*T*_emul_)

A single dose of the prepared Mgf-SPNMS (1 g) was poured drop-wise in 250 mL of 0.1 N HCl, while stirring at 50 rpm, using a USP XXXI Apparatus II (DS 8000, M/s Lab India Instruments, Mumbai, India) at ambient temperature. The emulsification time was assessed visually and medium was observed for self-emulsification in triplicate.

#### Globule size (*D*_nm_) and zeta potential

SPNMS were prepared by dilution (1 mL) in triplicate for *D*_nm_ and zeta potential analysis employing Zetasizer ZS 90, (M/s Malvern Instruments, Worcestershire, UK [42].

#### In vitro dissolution studies

The dialysis bag method was employed to study the in vitro release of Mgf from Mgf-SPNMS. Dissolution studies on the SPNMS formulation (*n* = 6), incorporated in hard gelatin capsules, were conducted in 250 mL of 0.01 N HCl containing 0.5% SLS using USP 31 Type II Apparatus, stirred at 50 rpm at ambient temperature. An aliquot of 5 mL each of the dissolution medium was withdrawn periodically, and replaced with fresh medium to maintain the sink conditions; analysis of Mgf was carried out at a λ_max_ of 262 nm using the HPTLC technique. From the drug release profile, CQAs like drug release in 15 min (Rel_15min_), mean dissolution time (MDT), and dissolution efficiency (DE) were calculated.

#### Transmission electron microscopy (TEM)

The formulation was observed for microscopy by placing it on a copper grid, stained with phosphotungstic solution (1%) for 30 s (JEM-2100 F, M/s Jeol, Tokyo, Japan).

#### Ex vivo permeation studies

The method has already been elaborated in one of the published works reported previously by our group [43]. The permeation studies were carried out by excising out the small intestine of sacrificed rats, washed with Kreb’s Ringer Buffer (KRB). The jejunum portion was everted on a glass rod after ligation with a thread and equilibrated subsequently in thermoregulated KRB solution. An accurately weighed amount of pure Mgf and optimized Mgf SPNMS (30 mg Mgf) were placed in bath medium outside the gut sac. From the gut sac, aliquots of samples (of 1 mL each) were periodically withdrawn and analyzed using HPTLC to discern the percentage of drug permeated in 45 min (Perm_45min_).

### MCF-7 and MDA-MB-231 cell-based testing

#### Cell culture

Human breast adenocarcinoma cells (MCF-7) were obtained from the University of Manchester, UK, and MDA-MB-231 cell lines were purchased from the European Collection of Authenticated Cell Cultures (ECACC), Public Health England, Salisbury, England. To grow MCF-7 cell lines, Dulbecco’s Modified Eagle’s Medium (DMEM, Sigma) and tissue culture flasks were employed (75 cm^2^) and maintained constantly in an incubator at 37 °C with 5% CO_2_. The culture was trypsinized using 1% trypsin, once the cells were 90% confluent. Similarly, to grow MDA-MB-231 cells, L-15 Medium (Leibovitz) was employed at 37 °C without CO_2_.

#### Cell viability assay

The concentration- and time-dependent cell viability assay of Mgf, blank SPNMS, and Mgf SPNMS were assessed on MCF-7 and MDA-MB-231 cell lines using PrestoBlue cell viability reagent (Invitrogen, USA). Briefly, an aliquot of 100 μL of medium containing 1000 cells per well was seeded in 96-well cell culture plates (Costars, Corning Inc., NY, USA) and incubated for a period of 24 h. The medium was replaced with 90 μL of different concentrations (10–1000 nM) of the said formulations. Cells were treated with the formulations in separate culture plates before incubating for 24, 48, and 72 h at 37 °C on 5% CO_2_. Before calculating the fluorescence, 10 μL PrestoBlue was added 1 h before and the culture plates were incubated at the end of respective time interval [39, 44]. Cell viability was determined using fluorescence measurement at excitation and emission wavelengths of 560 and 590 nm, respectively, and expressed as percentage normalized to untreated controlled cells.

#### Qualitative and quantitative cellular uptake

Investigations for qualitative cellular uptake were conducted by fluorescence microscopy on MCF-7 and MDA-MB-231 cell lines employing Rhodamine 123 (Rh-123) as a tracker dye, loaded in SPNMS [45]. Cells (1 × 10^5^ per well), both for MCF-7 and MDA-MB-231, were plated onto glass cover slips in DMEM and L-15 media, respectively, and allowed to adhere for overnight. Once adhered, the cells were treated with 0.064 μM Rh-123-loaded SPNMS for 15 min to 4 h separately at 37 °C. Pre-warmed PBS was used to wash cells thrice, subsequently fixed for 20 min at room temperature using 4% (*v*/*v*) paraformaldehyde. Cells adhering to microscopic slides were washed thrice with PBS prior to mounting with Vectashield®, a mounting medium containing 300 nM of DAPI (4′,6-diamidino-2-phenylindole), a fluorescent stain for nucleus staining. Cells were imaged on a modified cell observer imaging system (Zeiss EC Plan-Neofluar × 40/1.3 oil objective). Rh-123 and DAPI were imaged using a GFP/DAPI filter set with Ex/Em wavelength of 450–490 nm/500–550 nm and 335–383 nm/420–470 nm, respectively. Analysis of the images was carried out using the Zeiss ZEN desk imaging software. For quantitative measurement, cells (1 × 10^5^) were seeded in each well of six-well plate and incubated for overnight. Rh-123-loaded SPNMS (100 μg/mL) were added to each well of the six-well plate and incubated for different time intervals, in a manner similar to qualitative measurement [44, 46]. After respective time points, medium containing SPNMS was removed and cells were trypsinized and re-suspended in PBS for immediate flowcytometry analysis. Rh-123 signals were detected in FL-1 channel of BD FACSAria flowcytometer. A total of 10,000 events were processed and data were analyzed on Flowing version 2.5.1 (PertuuTerho, Turku Centre for Biotechnology, University of Turko, Finland).

#### P-gp efflux assay

Overexpression of P-gp in MCF-7 and MDA-MB-231 cells is well-documented in literature [47–49]. P-gp efflux pump does not let anticancer drugs to accumulate within the cell by effluxing it time and again, thereby preventing their cytotoxic or apoptotic effects on cancer cells. For evaluating the P-gp efflux, the multi-drug resistance dye efflux assay kit (Chemicon International, USA) was employed in which the MCF-7 and MDA-MB-231 cells (2.5 × 10^5^) were treated with Rh-123 and DiOC_2_ dyes, with or without vinblastine. To assess the inhibitory activity of SPNMS on MDR1 transporters, these were incubated with Rh-123 alone, Rh-123 with SPNMS, and Rh-123 with vinblastine at 37 °C, along with Rh-123 at 4 °C. Likewise, to estimate the potential of the developed SPNMS formulation for blocking the activity of BCRP transporters, DiOC_2_ dye was employed. On similar heels, MCF-7 and MDA-MB-231 cells (2.5 × 10^5^) were incubated with DiOC_2_ alone, DiOC_2_ with SPNMS, and DiOC_2_ with vinblastine at 37 °C, along with DiOC_2_ at 4 °C. The fluorescence intensity was measured in a TECAN fluorescence microplate reader at an excitation wavelength of 485 nm and an emission wavelength of 530 nm [50].

### Animal Studies

#### Animals

Animal studies were carried out on Sprague Dawley (SD) rats in bred and housed in Central Animal House, Panjab University, Chandigarh, India. Standard housing conditions were maintained and animals were kept on regular solid feed and water ad libitum. The animal experiments were performed in accordance with the recommendations of the committee for the purpose of control and supervision of experiments on animals (CPSCEA), India. The study protocol was approved by the institutional animal ethics committee (IAEC) of Panjab University, Chandigarh (Protocol no. 578/IAEC dated 1/08/2016).

#### Intestinal permeation study of Mgf: Confocal laser scanning microscopy

Small intestine from the abdominal cavity of SD rats (*n* = 3) was excised after sacrificing the rat and flushed it with 0.9% sodium chloride solution to remove any traces of feces and blood. Rh-123-loaded SPNMS formulation was introduced in the intestine and kept for 2 h [51]. Microm HM 525 U Cryostat, Thermo Fisher Scientific, USA, was employed for the study with a microtome, i.e., a slicer and a freezer. The intestine specimen was placed on a metal tissue disc, embedded in OCT, consisting of polyethylene glycol and polyvinyl alcohol, secured in a chuck, and frozen (− 20 to − 30 °C) rapidly to slice up to 10-μm sections. The section was placed on a glass slide mounted with glycerol, covered with a cover slip to capture the image with the confocal microscope (CLSM) (NIKON C2 PLUS, software IVIS Elements AR).

#### In situ intestinal perfusion

The in situ perfusion studies were performed as per the procedure previously reported in literature [52]. Briefly, unisex SD rats were put on fasting overnight and divided into two groups with three animals per group, viz. plain Mgf and Mgf-SPNMS. Anesthetized animals were slit open at their abdomen and their jejunum was incised at the upper and lower parts for 4 cm to perfuse KRB. An aliquot of perfusate (1 mL each) was withdrawn periodically and the content of Mgf was analyzed using the HPTLC analysis at 262 nm [37]. Various absorption and permeability parameters were calculated as per the details mentioned in Supplementary Section 2A.

#### In vivo pharmacokinetic studies

In vivo pharmacokinetic studies were conducted by sparse sampling designed experiment. The formulations were administered through oral gavage on two groups of rats (*n* = 60). Within each group, five subgroups were designated (5 × 6 = 30 animals were employed in one group) and from one animal maximum two blood samples were withdrawn during the entire study alternating between left and right eyes. Group I was administered plain Mgf solution and Group II was given Mgf-SPNMS containing Mgf equivalent to 30mg/Kg [39]. The animals were anesthetized employing isoflurane, and the blood (200 μL) was withdrawn from the retro-orbital plexus of rats at 0.5, 1, 2, 3, 6, 12, 18, 24, and 36 h, in heparin-coated micro-centrifuge tubes.

Further, plasma was harvested by centrifugation at 10,000 rpm (559 g) for 5 min and analyzed for Mgf using the previously reported and validated HPTLC method [37]. The data were analyzed by non-compartmental and compartmental pharmacokinetic modeling approaches using the WinNonlin software, version 5.0 (M/s Thermo Scientific, USA) [53]. Statistical tests like one-way/two-way ANOVA followed by post hoc multiple comparison tests, and Student’s independent *t* test has been applied wherever applicable.

#### Histopathology and hematological studies

A total of 12 SD female rats were divided into four groups, with three animals in each group. Plain Mgf, blank SPNMS, and Mgf-SPNMS (containing an equivalent amount of 30 mg/kg Mgf) were administered three times per week for 4 weeks in total by oral gavage with suitable intubation cannula. Animals of control group were administered normal saline. At the end of the study, the animals were euthanized by isoflurane. All the vital organs were excised, and fixed in 10% formalin for a minimum period of 24 h. Further, the tissues were passed through a cascade of steps like dehydration with increasing score of ethyl alcohol, clearing in xylol and mounting in molten paraplast at 58–62 °C [54], staining of sections of 5 μm with hematoxylin and eosin stain (H&E), and subsequent observation for any histopathological change(s) vis-à-vis control under a light microscope. The further procedural details of the histopathology are in Supplementary Section 2B.

Further, after anesthetizing the rats, the blood was collected into 10-ml citrate-phosphate-dextrose anticoagulant vacuum tubes (Haematologic Technologies Inc., USA) by cardiac puncture. Harvested blood was centrifuged (2200×*g*, 10 min at 4 °C), and the plasma and buffy coat were removed by aspiration. The thin smear slide of the blood from each group was observed under light microscopy. The further procedural details of the hematology are in Supplementary Section 2C.

## Results and discussion

### Risk Assessment Studies

Supplementary Fig. 1 displayed the Ishikawa diagram with the aim to identify CQAs of Mgf-SPNMS, constructed with the help of the Minitab 16 software; FMEA (Supplementary Table 4) was carried out to estimate the risk(s) caused by selected MAs and/or PPs.

### Preliminary Screening

The equilibrium solubility studies revealed that among the surfactants, maximum solubilized fraction of Mgf, i.e., 17.64 mg.mL^−1^, was observed in vitamin E TPGS (Supplementary Fig. 2A). Among the cosolvents, the highest solubility of Mgf was observed in PEG 200 (i.e., 10.23 mg.mL^−1^) (Supplementary Fig. 2B). For better solubilization of the drug in SPNMS, proper selection of emulgent and cosolvents is quite imperative, and their emulsification properties are based upon the reduction in energy required to emulsify [55]. The cosolvents further help in reduction of interfacial tension and formation of very fine droplets [56]. Further, it was evident from the ternary diagrams that the *S*_mix_ (3:1) formed a wider nanoemulsion region, as illustrated in Supplementary Fig. 3(A–D).

### Factor screening studies

Pareto charts and half-normal plots revealed the effect of MAs/PPs on the studied CQAs (i.e., Rel_15min_, *D*_nm_, and *T*_emul_) divulging that vitamin E TPGS (emulgent) and PEG 200 (cosolvent) were highly influential, as their effect was beyond *t* value and Bonferroni line limits (Supplementary Fig. 4(A–F)). Emulgent was found to exert a notable effect on the Rel_15min_, *D*_nm_, and *T*_emul_, while coemulgent and cosolvent had higher influence on *D*_nm_, and *T*_emul._ Nevertheless, the other employed factors like type of mixing, stirring speed, stirring time, and temperature were kept as constant for further studies, as these caused only a trifling effect on the studied CQAs.

### QbD-based model development and response surface analysis

The MLRA technique was employed to build second-order quadratic model and the coefficients for each of the CQAs [57] (Eq. 1). Excellent fit of the data was quite apparent from high values of coefficient of correlation, ranging between 0.965 and 0.998 (*p* < 0.001 in all the cases).

1 $$ Y=\kern0.5em {\beta}_1{X}_1+{\beta}_2{X}_2+{\beta}_3{X}_3+{\beta}_4{X}_1{X}_2+{\beta}_5{X}_1{X}_3+{\beta}_6{X}_2{X}_3+{\beta}_7{X}_1{X}_2{X}_3+{\beta}_8{X}_1{X}_2\left({X}_1-{X}_2\right)+{\beta}_9{X}_1{X}_3\left({X}_1-{X}_3\right)+{\beta}_{10}{X}_2{X}_3\left({X}_2-{X}_3\right) $$

where, *Y* is the response variable, *β*_1_ to *β*_4_ are the coefficients of linear model terms, *β*_5_ to *β*_7_ are the coefficients of quadratic model terms, *β*_8_ to *β*_10_ are the coefficients of cubic model terms with added interaction terms, while *X*_1_, *X*_2_, and *X*_3_ represent the factors employed (Supplementary Table 5).

**Fig. 1:**
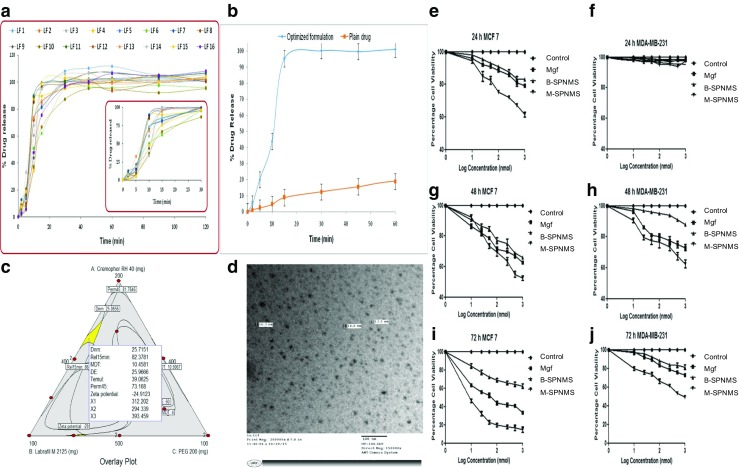
**a** Cumulative in vitro drug release profile of Mgf from various formulations prepared as per the I-Optimal mixture design. **b** In vitro drug release profile of optimized SPNMS and drug suspension of Mgf, values expressed in mean ± SD (*n* = 6). **c** Design space overlay plot for optimized SPNMS. **d** TEM images of SPNMS. Time- and dose-dependent percentage cell viability by different concentrations of Mgf, B-SPNMS (blank SPNMS), and M-SPNMS (Mgf-loaded SPNMS) at **e**–**f** 24 h, **g**–**h** 48 h, and **i**–**j** 72 h in MCF-7 and MDA-MB-231 cell lines. Data shown are mean ± SD from three independent experiments

The generated response surface plots helped in inclusive understanding of the impact of CMAs on the studied CQAs. The 3D-response surface plot in Fig. 1a indicates combined influence of all the CMAs, i.e., Phospholipid 90G, vitamin E TPGS, and PEG 200 on the studied CQA, i.e., globule size. A trivial increase in *D*_nm_ was observed at the high levels of Phospholipid 90G and intermediate levels of vitamin E TPGS, while a decreasing trend was noticed at the high levels of PEG 200. The minimum value for globule size was noticeable at the intermediate to high levels of vitamin E TPGS, and the intermediate levels of PEG 200, respectively. Smaller globule size, as is well-documented, is highly desirable for quicker absorption of drug into the systemic circulation [58].

Figure 1b depicts an umbrella-like 3D-response surface plot, portraying the maximum value of Rel_15min_ at the intermediate levels of Phospholipid 90G, vitamin E TPGS, and PEG 200. The plot shows a sharp rise in Rel_15min_ values at the intermediate levels of PEG 200 and vitamin E TPGS [59], indicating the highest values of Rel_15min._ Phospholipid 90G showed a negative effect on the release behavior, which could be attributed to its drug solubilization potential.

Figure 1c represents the 3D diagrammatic depiction of the positive influence of intermediate levels of Phospholipid 90G and vitamin E TPGS on the MDT values. However, on increasing levels of PEG 200, the values of MDT exhibited a declining trend. This unraveled a remarkable influence of lipids and surfactant on MDT, while the cosolvent had varying influence from negligible to high.

The 3D-response surface plot (Fig. 1d) exhibits a sharp decline with increase in the concentration of Phospholipid 90G and PEG 200, while vitamin E TPGS shows increase in the values of DE at its high level. Minimum value of DE was observed at the intermediate levels of vitamin E TPGS and PEG 200, and low levels of Phospholipid 90G.

Figure 1e shows negligible influence of all the constituents on the values of *T*_emul_, thus delineating the absence of interaction effect among them. At all the levels of the studied constituents, no significant change was observed (*p* > 0.05).

Figure 1f shows that at the lower levels of Phospholipid 90G, Perm_45min_ values were observed to be maximal, followed by decreasing trend with increased levels of vitamin E TPGS. On the contrary, PEG 200 shows declining effect on Perm_45min_ values. A hump-shaped curve in the 3D plot was observed at the intermediate levels of Phospholipid 90 G, indicating high values of Perm_45min_. This clearly supported the fact that Phospholipid 90G helps in faster permeation of drug across the GI tract into the systemic circulation.

Figure 1g exhibit declining values of zeta potential at increasing concentrations of Phospholipid 90G and PEG 200, while vitamin E TPGS shows increase in the zeta potential values at higher levels, followed by a dip at the highest levels. Higher values of zeta potential, required for maintaining physical stability of the nanoformulations, were observed at low to intermediate levels of all the constituents.

### Characterization of the prepared Mgf SPNMS

#### Globule size (*D*_nm_), zeta potential, and self-emulsification time (*T*_emul_)

The value of *D*_nm_, ranging between 15 and 60 nm, assures the nanomicellar nature of the developed formulations. Emulgents enable to reduce the interfacial tension and stabilize the globules, thus resulting in the formation of smaller globules [60]. Further, nanometeric size range of globules can be ascribed to the ability of the cosolvent to facilitate the efficient emulsification and fluidization of the oil-surfactant blend [61]. Zeta potential was found to range between − 20 and − 29 mV, indicating the stability of the developed formulation. It is reported in literature that the negative charge in nanomicelles is due to the presence of free fatty acids on oil droplets [62]. *T*_emul_ values were observed to range within 22 to 64 s. Less values of *T*_emul_ indicated the tendency of the formulation to emulsify faster in order to produce transparent nanoemulsion [53].

#### In vitro drug release studies

From the dissolution studies, nearly complete drug release (> 90%) was observed for all formulations resulted within 1 h indicating faster Mgf release from SPNMS (Fig. 2a). The inset in the figure is a clear testimony to more than 80% of Mgf release in 15 min. Also, significant improvement (i.e., about fivefolds) in the dissolution rate was observed vis-à-vis free Mgf suspension (*p* < 0.001) (Fig. 2b). MDT was found to vary between 3 and 18 min, while %DE was found to vary between 22 and 34%.

**Figure:**
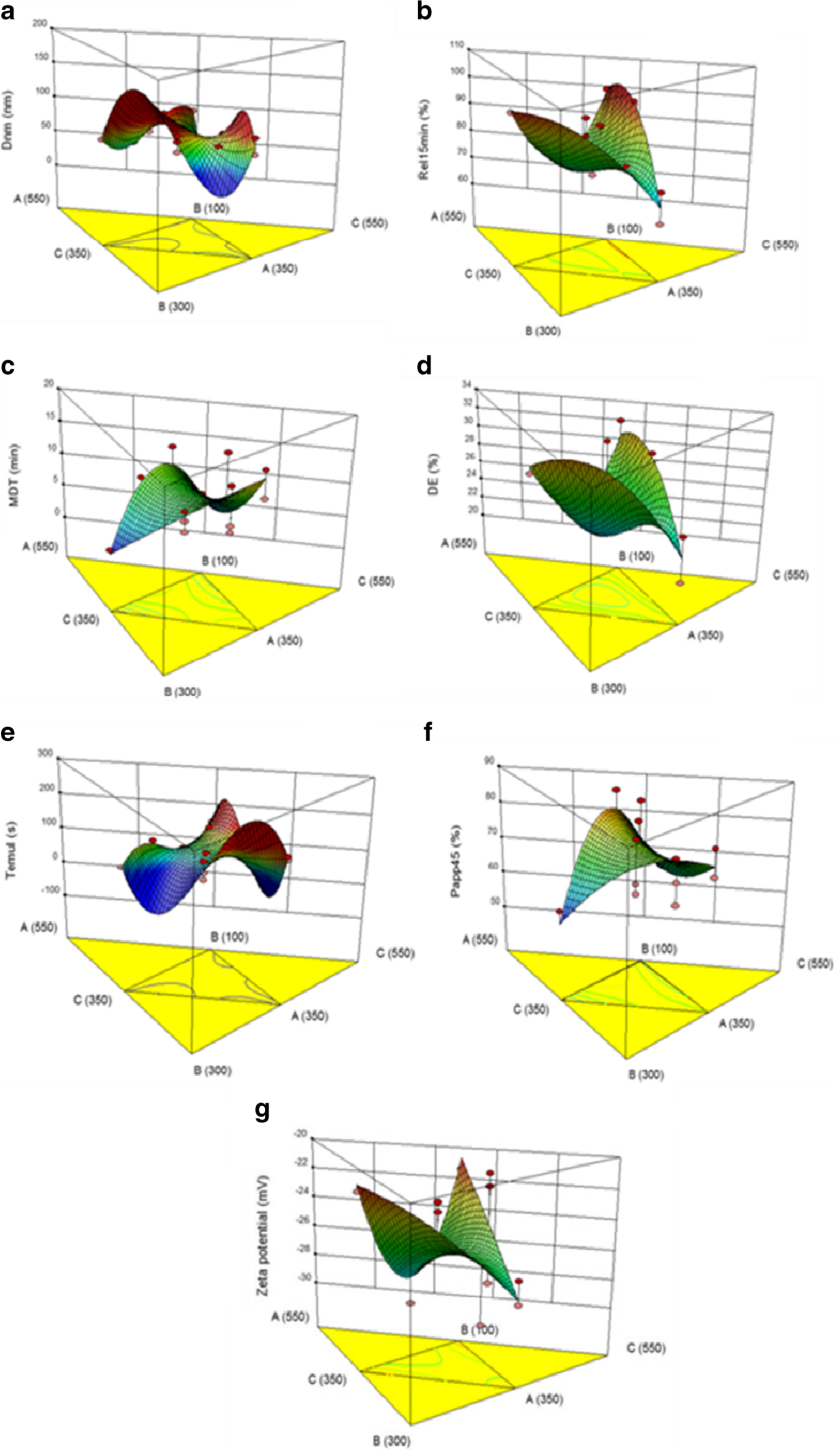

#### Permeability studies

Everted gut sac studies indicated that the values of intestinal permeability varied from 59 to 88% for all the developed SPNMS of Mgf. The results are in consonance with literature reports, where the authors also report the increase in membrane permeability with self nano-emulsifying globules [63–65].

### Search for the optimum formulation and validation of QbD

Selected CQAs were traded-off to arrive at the optimum formulation and to achieve requisite objectives, i.e., smaller *D*_nm_, minimal *T*_emul_, highest Rel_15min_, and maximum Perm_45min_. Therefore, the selection criteria were finalized to search the optimized formulation with *D*_nm_ < 50 nm, zeta potential <− 30, *T*_emul_ < 1 min, Rel_15min_ > 80%, DE > 20%, MDT > 7 min, and Perm_45min_ > 70%. Numerical desirability methodology was adopted for identifying the optimum formulation, where all the CQAs exhibited the value of desirability function close to unity. Figure 2c portrays the optimized SPNMS of Mgf, demarcated in the design space overlay plot. The optimized formulation contained Phospholipid 90G (312 mg), vitamin E TPGS (294 mg), and PEG 200 (393 mg), with the values of CQAs as *D*_nm_ of 25 nm, zeta potential of − 25, *T*_emul_ of 39 s, Rel_15min_ of 82%, DE of 26%, MDT of 10, and Perm_45min_ 73%, respectively. The TEM analysis of the optimized formulation (Fig. 2d) evidently indicated spherical globules of the SPNMS formed with a mean size of about 15 nm.

### Cell line results

#### Cell viability assay

Various percentage cell viability plots portray that Mgf-SPNMS show the higher toxicity towards MCF-7, suggesting faster onset of action in 24 h, whereas hardly any cell death was observed for MDA-MB-231 cells at the same time-point. Maximum cell death for MDA-MB-231 cells at similar concentration ranges was observed at 72 h. Also, IC_50_ value for MCF-7 cells was found to be 37.56, 22.65, and 4.37 nM at 24, 48, and 72 h, respectively, while negligible cell death was observed at 24 h and a slightly higher IC_50_, i.e., 52.13 and 45.39 nM, was observed with MDA-MB-231 at 48 and 72 h, respectively (Fig. 2e–j). This could be attributed to the higher accumulation of Mgf-SPNMS in MCF-7 than MDA-MB-231 cells, which is known to express P-gp efflux transporters. The higher expression of P-gp receptors on MDA-MB-231 might be responsible for lower accumulation of Mgf-SPNMS in these cells [66]. Moreover, triple negative MDA-MB-231 cells, which bear an aggressive phenotype, usually respond less favorably to the compounds than the less aggressive, estrogen receptor positive, MCF-7 breast cancer cells [67, 68].

#### Qualitative and quantitative cellular uptake

**Fig. 3:**
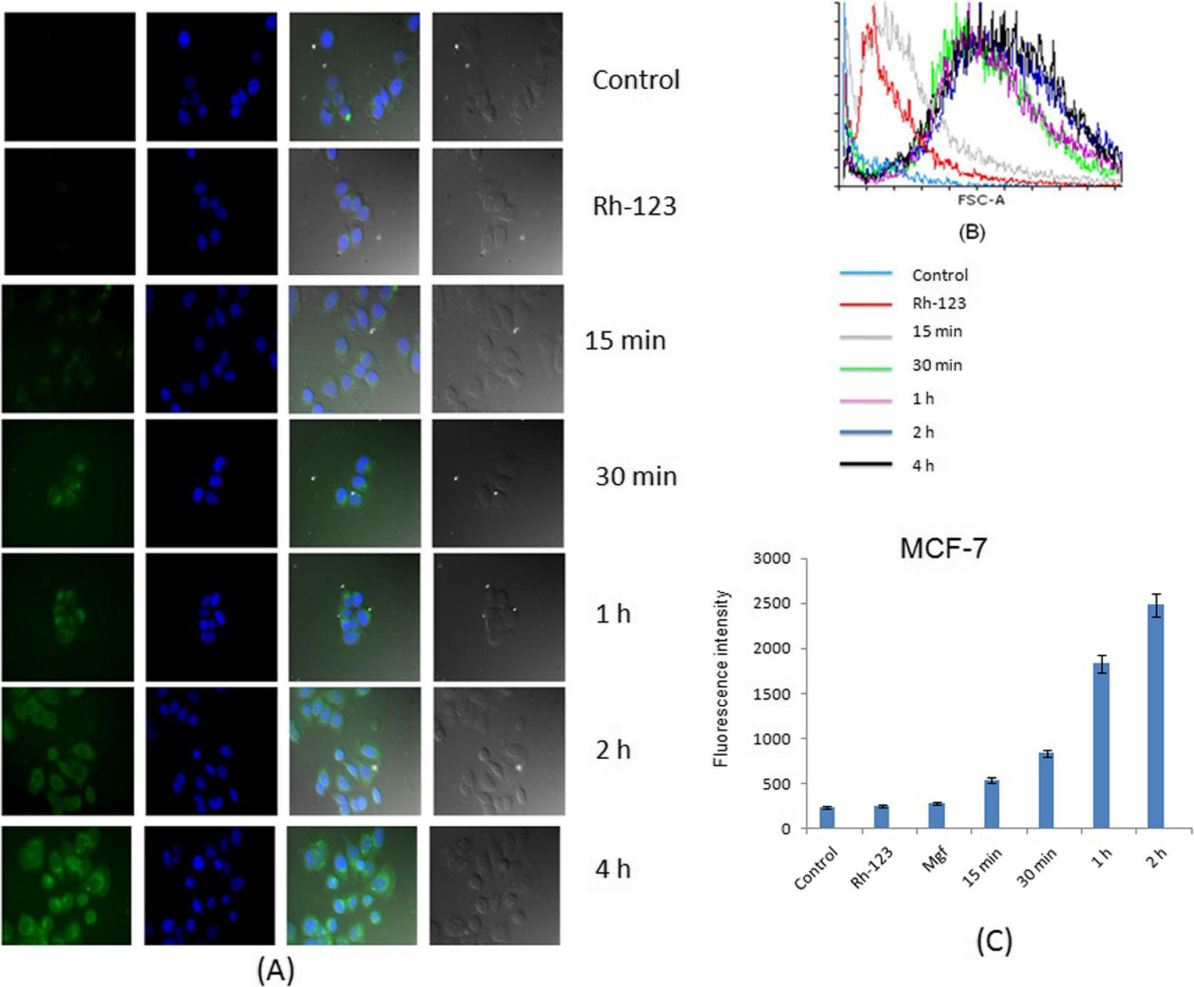

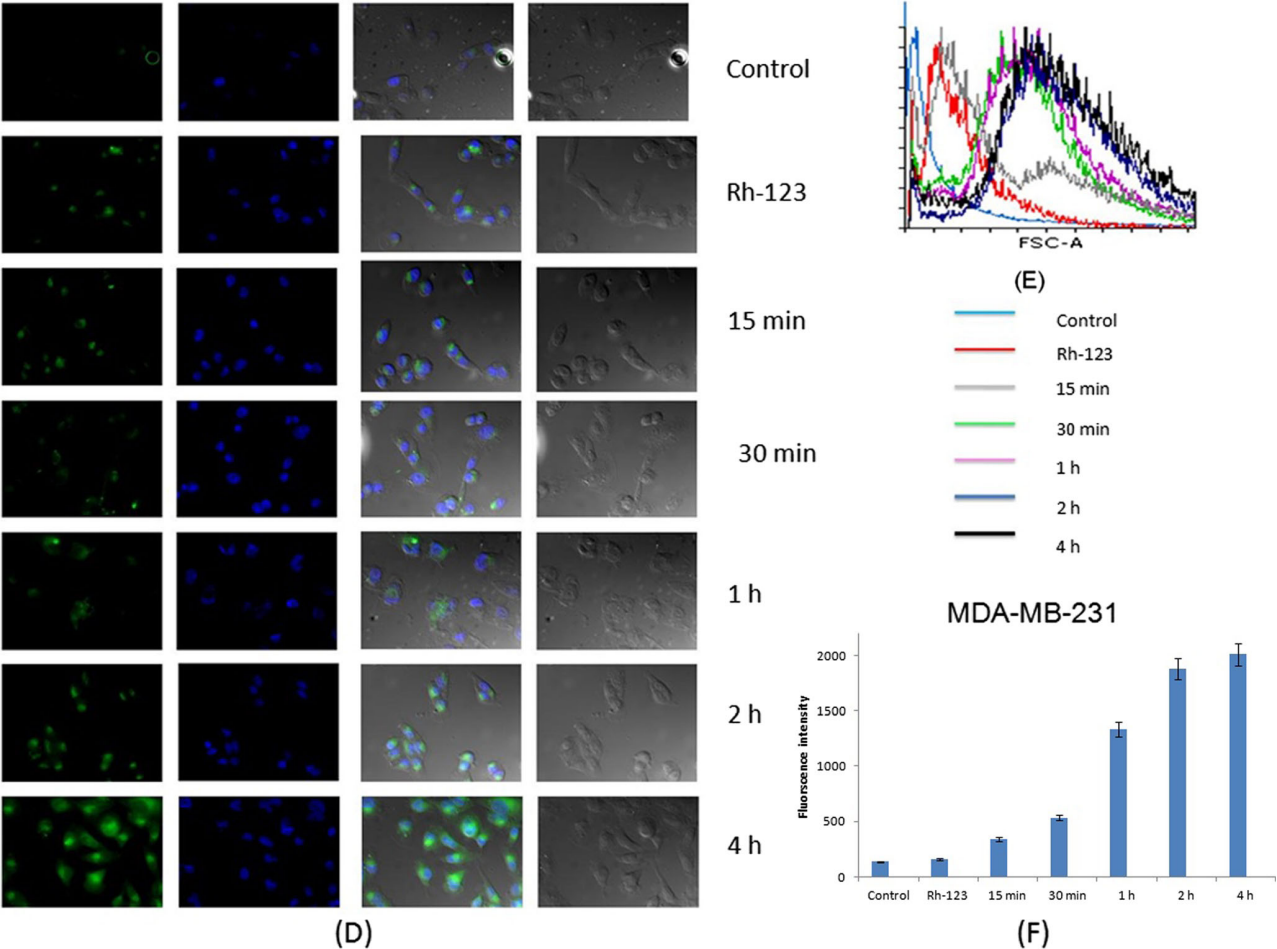
**a** and **d** Qualitative analysis indicated the increased cellular uptake with the increase in the Rh-123 intensity for MCF-7 and MDA-MB-231, respectively. **b** and **e** Flow cytometry histogram overlays for MCF-7 and MDA-MB-231 cells of Rh-123 SPNMS following control (untreated cells), 15 min, 30 min, 1 h, 2 h, and 4 h incubation at 37 °C. **c** and **f** Fluorescence intensity plotted vs. time also corroborated the cellular uptake for MCF-7 and MDA-MB-231, respectively

On investigating the cellular uptake on MCF-7 and MDA-MB-231 cells, it was observed that Rh-123-SPNMS started migrating within both the cells in 15 min. Qualitative analysis made through the images were processed through Zen Pro 2012, displaying the fluorescent images of Rh-123-SPNMS uptake at various time-points (Fig. 3a, d). The corresponding overlay histograms (Fig. 3b, e), generated after the data were analyzed using the Flowing software, further corroborated the substantial cellular uptake of Rh-123-SPNMS at the same time points, i.e., 15, 30 min, and 1, 2, and 4 h. Through the qualitative and quantitative observations made, intensity of fluorescence was observed to be much higher for MCF-7 cells than for MDA-MB-231 cells (*p* < 0.05) (Fig. 3c, f), suggesting that the SPNMS encountered less hindrance with the former cell lines.

Therefore, one could conclude and correlate the cellular viability and uptake results, in which higher and quicker cellular uptake of the formulation is the indicative of the lower IC_50_ values obtained for both the cell lines. Also, it is reported that the higher cellular uptake of the SPNMS is owing to the presence of surfactants, attributing to the enhancement of the permeability and could change the cell integrity [69, 70].

#### P-gp efflux assay

**Fig. 4:**
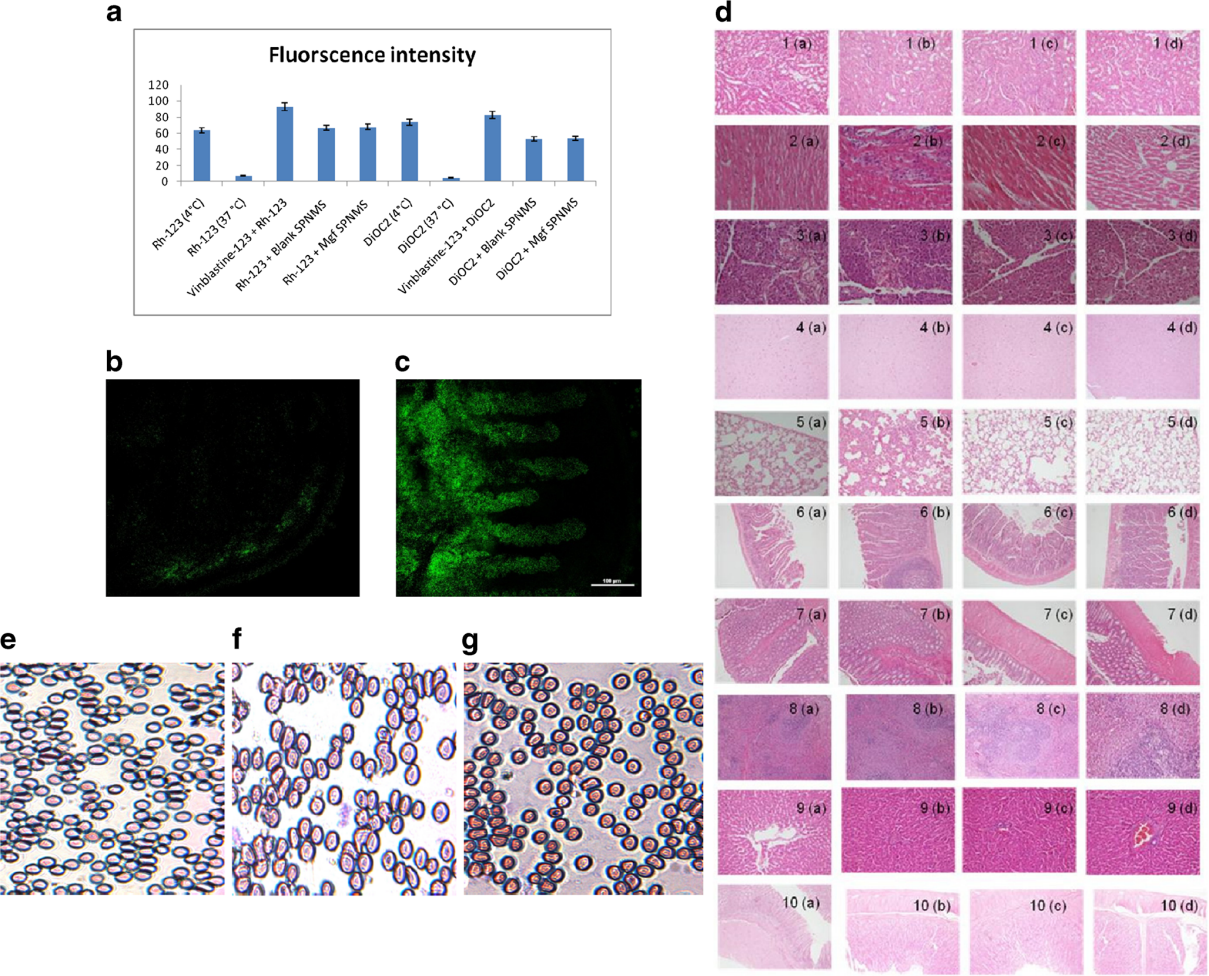
**a** Relatively higher fluorescence intensity index indicates inhibition of MDRI and BCRP transporters maximally by Mgf-SPNMS loaded with Rh-123 and DiOC_2_ dye, respectively. **b** Shows no migration of Rh-123 dye into the intestine. **c** Rh-123 loaded SPNMS has migrated to microvilli of intestine, indicating its better absorption. **d** Histopathological findings were examined on comparing with the (a) untreated rat after administering saline solution, (b) plain Mgf, (c) blank SPNMS formulation, and (d) Mgf SPNMS. (1) Kidney (a, b, c, d): glomeruli, tubules, and blood vessels are within normal limits. (2) Heart (a, b, c, d): endocardium, epicardium, and myocardium do not show any significant changes. (3) Pancreas (a, b, c, d): pancreatic acini and islets do not show any significant changes. (4) Brain (a, b, c, d): the meninges, cerebral cortex, white matter, cerebellum, hippocampus, and choroid plexus are within normal limits. (5) Lung (a, b, c, d): the pleura, parenchyma, and the interstitium do not show any significant changes. (6) Small intestine (a, b, c, d): ileum shows normal villi and the brush borders are maintained. No other significant changes are observed. (7). Large intestine (a, b, c, d): mucosa, submucosa, muscularis propria, and serosa are within normal limits. (8). Spleen (a, b, c, d): the splenic red and white pulp does not show any significant changes and are within normal limits. (9) Liver (a, b, c, d): the portal tracts, central vein, and sinusoids do not show any significant changes. (10) Stomach (a, b, c, d): mucosa, submucosa, muscularis propria, and serosa do not show any significant changes. Pictures of whole blood extracted from rats after various treatments **e** control, **f** Mgf, and **g** Mgf SPNMS

The P-gp efflux assay revealed that at 4 °C, there was no evidence of active transport, as the MDR and BCRP transporters were inactive, leading to higher accumulation of dyes inside the cells, as can be clearly evident from the fluorescence intensity (Fig. 4a). On the other hand, incubation at 37 °C showed active efflux of both dyes (Rh-123 and DiOC2); however, in the presence of vinblastine, which blocks both the MRP2 and BCRP transporters, lead eventually to higher fluorescence intensity without any efflux [71]. Relatively less efflux of Rh-123 and DiOC2 in the presence of SPNMS could be attributed to the excipients like vitamin E TPGS [72] and PEG [73, 74], which act as the P-gp efflux inhibitors, which is in total agreement with the literature reports. In this context, SPNMS would be an adept carrier for delivery of Mgf, which is a P-gp substrate [75], facilitate its accumulation in cancer cells by weakening its P-gp mediated efflux.

### Intestinal permeation study of Mgf

Figure 4b shows that Rh-123 dye, of its own, does not permeate through the intestine, as it has a tendency to get effluxed by P-gp receptors. However, using optimized Rh-123 SPNMS (Fig. 4c), the fluorescent microvilli of the intestine were observed, indicating the penetration of the prepared formulation, majorly due to the droplets being formed in the nanometric size range. Consequently, the outcome from the CLSM study corroborates improved intestinal permeation with SPNMS, ostensibly owing to the formation of nano-micelles and the presence of vitamin E TPGS [72] and PEG to act as P-gp inhibitors [74].

### In situ intestinal perfusion studies

**Fig. 5:**
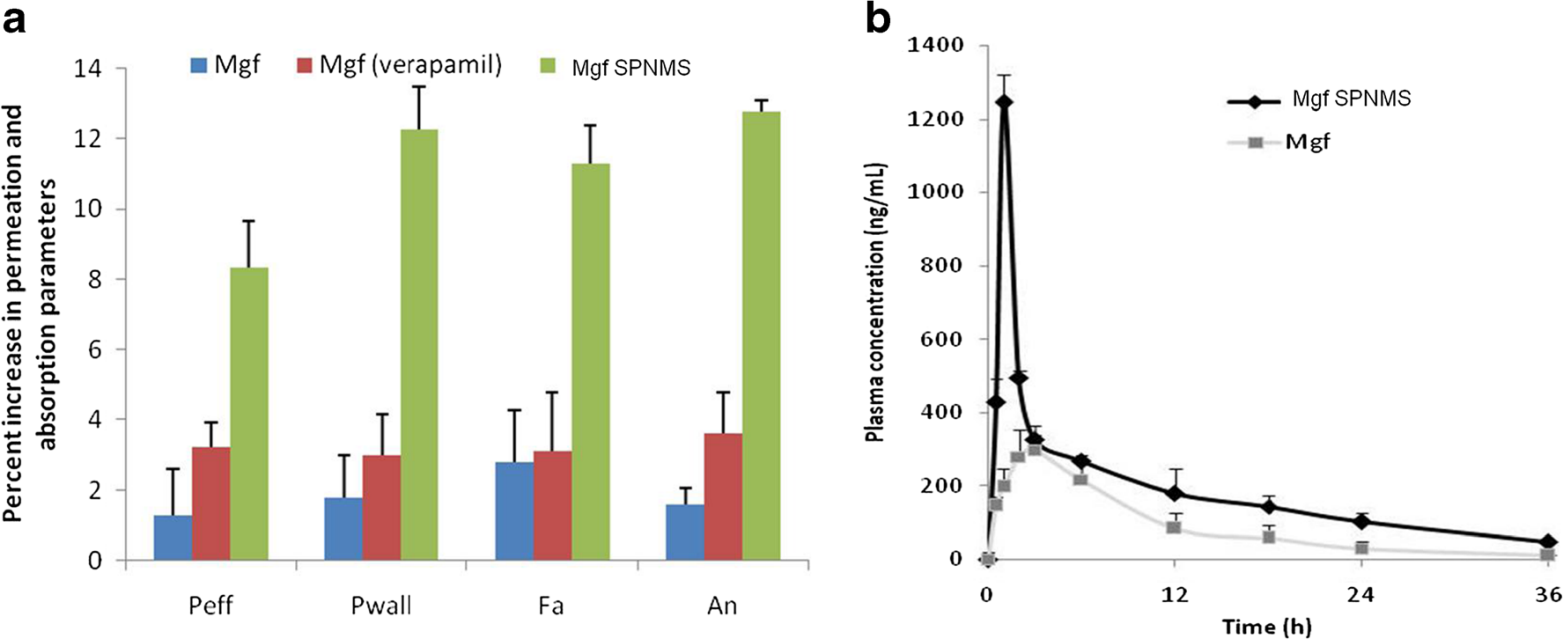
**a** Percent increase in permeability and absorption parameters of Mgf calculated after administering optimized Mgf SPNMS, Mgf administered with verapamil vis-à-vis pure Mgf. **b** Mean plasma concentration-time curve of Mgf and Mgf SPNMS equivalent to 30 mg/mL (*n* = 6), respectively; mean value of ± SD

These studies explore the absorption and permeation behavior of a drug, when administered as an oral formulation [24]. With SPNMS, enhanced values of absorptivity and permeability parameters of Mgf were observed vis-à-vis pure bioactive. As is evident from Fig. 5a, Mgf SPNMS showed significantly escalation in the values of (*p* < 0.001) absorption number (An) by 8.12-fold and 3.54-fold, vis-à-vis pure Mgf alone, and with verapamil, respectively. The amount of drug transferred across the GI tract, where Mgf SPNMS exhibited distinct superiority over free bioactive, indicated enhanced drug absorption characteristics [76].

Verapamil, being a potent P-gp inhibitor, was able to improve the permeation and absorption parameters, but only up to a limited extent [77, 78]. Further, a significant increase (*p* < 0.01) in the values of fraction drug absorbed (Fa) was also observed for Mgf SPNMS (i.e., 4.06-fold), and Mgf administered with verapamil (i.e., 3.64-fold) vis-à-vis pure Mgf. The aforementioned results indicated notable augmentation in the absorption potential of Mgf SPNMS, ostensibly due to transport of bioactive through lymphatic pathways via circumnavigation of its first-pass effect in the liver [24, 79, 80].

Similarly, in case of permeability parameters, the values of effective permeability (*P*_eff_) also showed significant, i.e., nearly 6.56-fold and 2.57-fold improvement with SPNMS of Mgf and Mgf-verapamil (*p* < 0.001 each) vis-à-vis pure Mgf, respectively. Likewise, significant increase (*p* < 0.001) in the corresponding values of wall permeability (*P*_wall_) was also observed by about 6.88-fold and 4.11-fold. The greater values of *P*_eff_ and *P*_wall_ delineate increase in the permeability and uptake characteristics of the optimized formulation, plausibly ascribed to improved permeability and decreased efflux due to the incorporation of emulsifying excipient in the formulations [81, 82].

### In vivo pharmacokinetic studies

Following per oral administration of Mgf SPNMS and pure Mgf, plasma levels were monitored in rat at the designated time-points. Thus, the pharmacokinetic profile (Fig. 5b) shows considerable difference in the plasma concentrations for Mgf SPNMS vis-à-vis pure Mgf (dose equivalent to 30 mg/kg [39]) at all the time-points studied (*p* < 0.001). Furthermore, a linear decline in the post-*T*_max_ log concentration-time profile showed that the drug profile follows 2-CBM kinetics [19]. Table 1 shows various calculated pharmacokinetic parameters for Mgf and Mgf SPNMS with statistically significant (*p* < 0.001) difference among all the parameters as estimated in rats treated with pure plant bioactive versus its SPNMS formulation.

**Table 1:** Pharmacokinetic parameters obtained from in vivo plasma level studies in rat following oral administration of Mgf and its SPNMS

Treatment formulations	Pharmacokinetic parameters						
	*C*_max_ (ng.h^−1^)	AUC_last_ (h × ng.ml^−1^)	*C*_max_/AUC (h^−1^)	Ka (h^−1^)	*T*_max_ (h)	MRT (h)	Cl (mL.h^−1^)
Mgf	297.14 ± 56.36	213.38 ± 25.13	0.098	0.32 ± 0.09	3.08 ± 0.18	8.16 ± 1.73	2543.44 ± 453.42
Mgf SPNMS	1254.62 ± 182.15	8962.15 ± 357.18	0.017	1.15 ± 0.25	1.43 ± 0.24	14.19 ± 1.65	1245.72 ± 267.13

All the data represented as mean ± SD (*n* = 3)

Nearly 4.22- and 2.96-fold increase in the magnitude of *C*_max_ and AUC was noted on comparing with pure drug (*p* < 0.001), respectively. Moreover, there was 2.65-fold reduction in *T*_max_ vis-à-vis pure Mgf (*p* < 0.001), thus corroborating quite faster onset of action owing to improvement in absorption rate. Besides, maximal variation was observed in the values of Ka, indicating enhanced oral drug absorption of Mgf SPNMS revealing nearly 3.59-fold increase in Ka over plain Mgf (*p* < 0.001). By and large, the pharmacokinetic studies in rats ratified the superiority of the formulated Mgf SPNMS in enhancing oral absorption of Mgf.

### In vivo toxicity studies

Figure 4d portrays no histopathological change(s) in all the vital organs, indicating that all the three treatments, i.e., Mgf (Fig. 4d (b)), blank (Fig. 4d (c)), and Mgf SPNMS (Fig. 4d (d)) were found to be quite biosafe as that of control (Fig. 4d (a)). Further, all the treated groups, i.e., Mgf, blank SPNMS, and Mgf-loaded SPNMS showed insignificant (*p* > 0.05) pathological changes vis-a-vis the control group.

The whole blood count analysis revealed that all the three treatments were not significantly (*p* > 0.05) affecting the white blood cells (WBC), red blood cells (RBC), hemoglobin (HGB), hematocrit (HCT), and platelet (PLT) count. Treatments with Mgf, blank SPNMS, and Mgf SPNMS decreased the values of WBC, RBC, HGB, and PLT, but the change was not found to be that profound (Table 2). The slides of the blood sample were prepared and observed under upright light microscope. Results showed no morphological changes in the shape of RBC, when treated with any of the formulation on comparing with the control (Fig. 4e–g). As it is evident from the histopathology studies (Fig. 4d) that the Mgf alone, blank SPNMS and Mgf SPNMS do not show any sign of toxicity on all the vital organs. The results are in accordance with the literature reports where the blank nanomicelles have shown practically no toxicity [83, 84].

**Table 2:** Whole blood count parameters obtained from blood of rats following various treatments of Mgf and its SPNMS

Mode	Control	Mgf	Blank SPNMS	Mgf SPNMS
	Count	Count	Count	Count
WBC	19.8 × 10^3^/μL	16.4 × 10^3^/μL	18.7 × 10^3^/μL	17.5 × 10^3^/μL
RBC	10.78 × 10^6^/μL	8.21 × 10^6^/μL	9.53 × 10^6^/μL	7.48 × 10^6^/μL
HGB	15.14 g/dL	14.54 g/dL	14.10 g/dL	13.32 g/dL
HCT	45.7%	43.9%	44.9%	42.6%
PLT	AG* 760 × 10^3^/μL	AG* 589 × 10^3^/μL	AG* 745 × 10^3^/μL	AG* 638 × 10^3^/μL

*All values are mean ± 3

## Conclusions

In the current studies, SPNMS of Mgf were systematically and successfully developed, which finally composed of Phospholipid 90G, vitamin E TPGS, and PEG 200. Apart from its surfactant like property, vitamin E TPGS was incorporated in the formulation to prevent any possible oxidation of Mgf, and its level was kept as constant throughout the study.

In this piece of work, in vitro, in situ, and in vivo studies carried out on the formulations proved these nanostructures to be highly superior to pure bioactive. The toxicity studies confirmed that the ingredients employed in the formulations were quite biosafe. Therefore, it can be concluded that SPNMS with high drug pay-load possess tremendous promise to augment oral bioavailability of Mgf and several other agents of BCS class IV marked with poor solubility and permeability characteristics like Mgf.

## Electronic supplementary material

ESM 1 (DOC 2066 kb)
