# Supplementary material for: Improving the biopharmaceutical attributes of mangiferin using vitamin E-TPGS co-loaded self-assembled phosholipidic nano-mixed micellar systems
Source: Drug Deliv Transl Res. 2018 Apr 10;8(3):617–32. doi: 10.1007/s13346-018-0498-4 (PMC5937873; doi:10.1007/s13346-018-0498-4)
Supplement: Supplementary file 1 — (DOC 2066 kb) [file 13346_2018_498_MOESM1_ESM.doc]

Supplementary Table 1: Quality target product profile (QTPP) for SPNMS of Mgf

| **QTPP Elements** | **Target** | **Justification(s)** |
| --- | --- | --- |
| Dosage category | Faster release | With better therapeutic benefits for quicker action |
| Dosage form | Liquid | Selection of water soluble emulgents would help in solubilisation of Mgf for better bioavailability |
| Dosage strength | 30 mg | Oral dose of the Mgf for treating cancer |
| Pharmacokinetics | C_max_, AUC | Achieve higher drug levels into the systemic circulation for enhanced therapeutic action |
| Route of administration | Oral | Most sought after route for delivery of drugs; more patient compliance and non-invasive |
| Stability | 24 months as per long term conditions | To ensure the constant potential of Mgf while storage |

Supplementary Table 2: Critical quality attributes (CQAs) for SPNMS of Mgf and their justifications

| **Quality attributes of drug product** | | **Target** | **CQA?** | **Justification(s)** |
| --- | --- | --- | --- | --- |
| Physical  attributes | Color | Acceptable to patients | No | Ensure the aesthetic acceptance of the product |
|  | Odor |  |  |  |
|  | Appearance |  |  |  |
| Emulsification time (T_emul_) | | Low | Yes | Lower T_emul_ enables the faster emulsification of formulation, thus enabling formation of nanomicelles |
| Assay and drug content | | 100% | No | Drug content and assay variability are the essential criteria to achieve uniform and maximum plasma concentration |
| Globule size (D_nm_) | | <50 nm | Yes | Lesser D_nm_ enables easier penetration of nanomicelles through GI tract by energy dependent endocytic pathways |
| Dissolution efficiency (DE%) | | Maximum | Yes | Indicator of the faster and complete drug release |
| Mean dissolution time (MDT) | | 100% | Yes |  |
| Apparent permeability in 45 min (Perm_45min_) | | ≥ 90% | Yes | Increased Perm_45min_ helps in enhanced absorption with better systemic circulation |
| Amount of drug release in 15 min (Rel_15 min_) | | 100% | Yes | Marker of faster drug release |

Supplementary Table 3: Summary of all the formulations prepared as per I-optimal mixture design for SPNMS of Mgf

| **Trials** | **Phospholipid 90G (mg)** | **Vitamin E TPGS (mg)** | **PEG 200**  **(mg)** |
| --- | --- | --- | --- |
| L-SPNMS 1 | 351.7 | 250.0 | 398.2 |
| L-SPNMS 2 | 297.9 | 347.9 | 354.0 |
| L-SPNMS 3* | 500.0 | 251.7 | 248.2 |
| L-SPNMS 4* | 500.0 | 400.0 | 100.0 |
| L-SPNMS 5* | 349.9 | 400.0 | 250.0 |
| L-SPNMS 3* | 500.0 | 251.7 | 248.2 |
| L-SPNMS 4* | 500.0 | 400.0 | 100.0 |
| L-SPNMS 6 | 426.3 | 173.6 | 400.0 |
| L-SPNMS 5* | 349.9 | 400.0 | 250.0 |
| L-SPNMS 7 | 402.1 | 301.4 | 296.3 |
| L-SPNMS 1* | 351.7 | 250.0 | 398.2 |
| L-SPNMS 8 | 448.1 | 352.2 | 199.5 |
| L-SPNMS 9 | 500.0 | 174.6 | 325.3 |
| L-SPNMS 5* | 349.9 | 400.0 | 250.0 |
| L-SPNMS 10 | 200.0 | 400.0 | 400.0 |
| L-SPNMS 11 | 500.0 | 100.0 | 400.0 |

*Formulations are prepared in duplicate

Supplementary Table 4: Abridged FMEA matrix enlisting the RPN scores for the selected product and process variables

| **S. No** | **Failure Modes** | **Unit** | **Severity**  **(S)** | **Occurrence**  **(O)** | **Detection**  **(D)** | **RPN** |
| --- | --- | --- | --- | --- | --- | --- |
|  | Type of emulgent | - | 8 | 5 | 3 | 120 |
|  | Type of cosolvent | - | 7 | 5 | 4 | 140 |
|  | Type of co-emulgent | - | 6 | 5 | 3 | 90 |
|  | Emulgent conc. | mg | 8 | 7 | 6 | 336 |
|  | Co-solvent conc. | mg | 6 | 6 | 6 | 216 |
|  | Co-emulgent conc. | mg | 7 | 6 | 5 | 210 |
|  | Order of Mixing | - | 4 | 4 | 5 | 80 |
|  | Stirrer Type | - | 4 | 5 | 4 | 80 |
|  | Stirring Speed | rpm | 6 | 6 | 6 | 216 |
|  | Stirring time | min | 7 | 5 | 5 | 175 |
|  | Temperature | °C | 8 | 6 | 5 | 240 |
|  | Cooling temperature | °C | 3 | 4 | 4 | 48 |


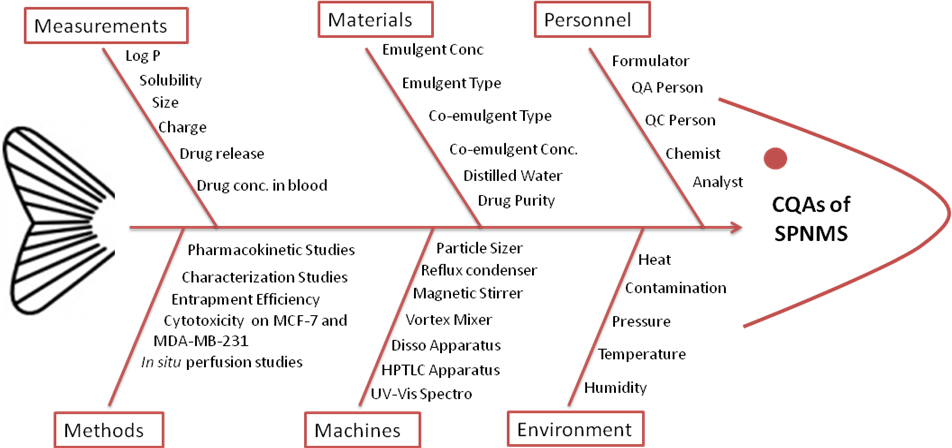


**Supplementary Figure 1: Ishikawa fish-bone diagram depicting the cause-and-effect relationship for SPNMS of Mgf.**


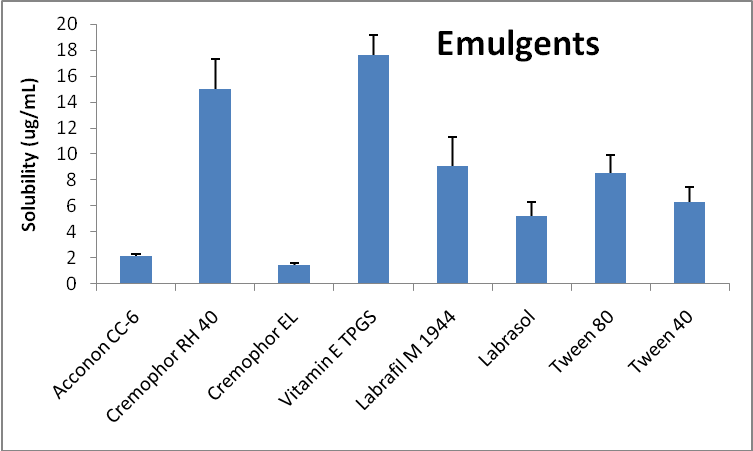


A


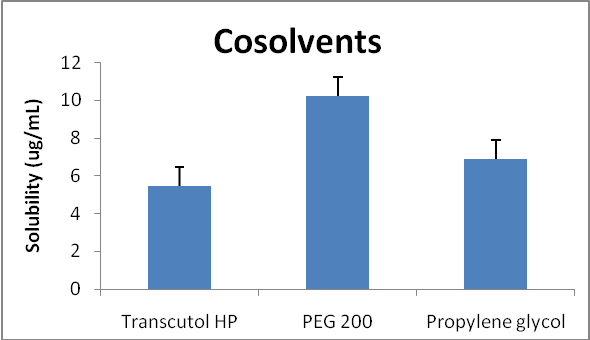


B

Supplementary Figure 2: Equilibrium solubility data of Mgf in various (A) emulgents and (B) cosolvents


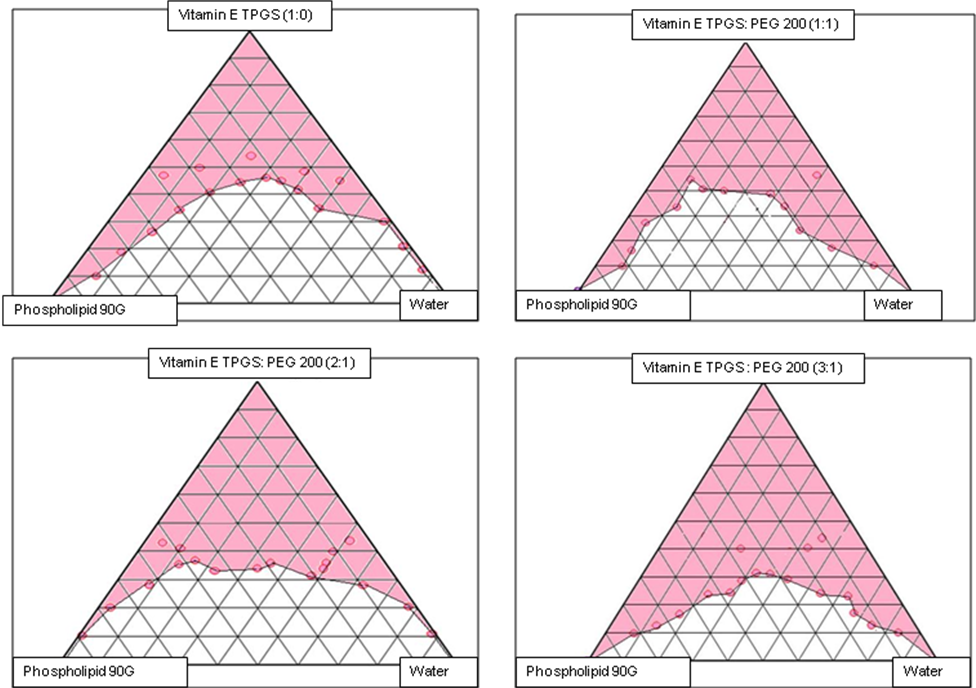


Supplementary Figure 3: Phase behavior of nanoemulsion formed from titration in the range of 1:9–9:1 for titrating Phospholipid 90G with S_mix_ (Vitamin E TPGS and PEG 200) ratios of (A) 1:0, (B) 1:1, (C) 2:1, (D) 3:1 and water


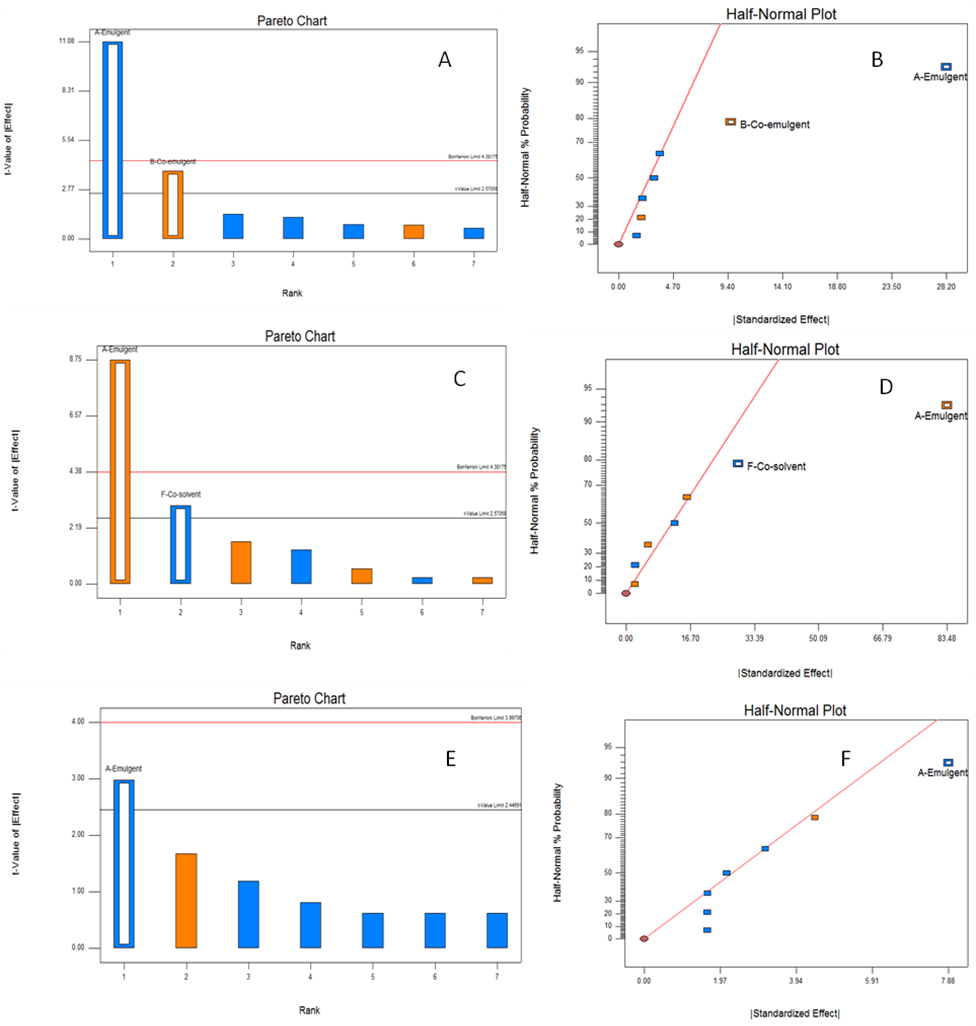


Supplementary Figure 4: Pareto and half-normal charts showing the influence of MAs/ PPs on the CQAs (A-B) Rel_15min_ (C-D) D_nm_, E-F) T_emul_

Supplementary Table 5: Values of polynomial coefficients and correlation coefficients for various CQAs of SPNMS of Mgf

| **Coef. code** | **Polynomial coefficients for response variables** | | | | | | |
| --- | --- | --- | --- | --- | --- | --- | --- |
|  | **D_nm_** | **Rel_15min_** | **DE_15min_** | **MDT** | **T_emul_** | **Perm_45min_** | **Zeta**  **potential** |
| β_1_ | -700.95 | 319.97 | 155.34 | 39.80 | 129.71 | 121.85 | -45.51 |
| β_2_ | -1142.10 | 478.69 | 218.97 | -8.08 | 1041.40 | 35.81 | 1.89 |
| β_3_ | 535.98 | 544.14 | 217.88 | -30.82 | -1984.12 | -41.43 | -1.71 |
| β_4_ | 4018.93 | -1203.77 | -625.58 | -53.79 | -1846.67 | -83.27 | 2.92 |
| β_5_ | 702.78 | -1332.34 | -624.25 | 3.58 | 4316.38 | 118.15 | 11.83 |
| β_6_ | 1560.23 | -1767.66 | -783.00 | 141.27 | 2603.43 | 316.53 | -110.64 |
| β_7_ | -4544.23 | 2899.02 | 1379.09 | -- | -4203.43 | -- | -- |
| β_8_ | -4543.31 | -- | -- | -- | 8596.29 | -- | -- |
| β_9_ | -- | -- | -- | -- | -9939.14 | -- | -- |
| β_10_ | -- | -- | -- | -- | 3366.00 | -- | -- |
| R | 0.9417 | 0.9995 | 0.9901 | 0.9565 | 0.9845 | 0.9917 | 0.9916 |
| P | < 0.001 | < 0.001 | < 0.001 | < 0.001 | < 0.001 | < 0.001 | < 0.001 |

- From the nature (positive or negative values) of coefficients, the type of interaction (synergism or antagonism) amongst the CMAs was discerned

**Supplementary Section 1:**

Initially, the quality target method profile (QTMP) was defined and critical analytical attributes (CAAs) earmarked, *viz.* retardation factor (R_f_), peak height, capacity factor, theoretical plates and separation number. Face centered cubic design (FCCD) was selected for optimizing of volume-loaded and plate dimensions as the critical method parameters (CMPs) selected from screening studies employing D-optimal and Plackett-Burman designs studies, followed by evaluating their effect on the CAAs. The mobile phase containing mixture of ethyl acetate: acetic acid: formic acid: water in 7:1:1:1, v/v/v/v ratio was finally selected as the optimized solvent for apt chromatographic separation of mangiferin at 262 nm with R_f_ 0.68±0.02 and all other parameters within the acceptance limits. Method validation studies revealed high linearity in the concentration range of 50–800 ng/band for mangiferin.

The optimum solutions was carried out by numerical optimization while “trading off” various CAAs for attaining optimum values of R_f_, peak height, separation number, theoretical plates and capacity factor, all indicative of the efficient densitometric separation and enhanced resolution of the mangiferin. The numerical optimization suggested CMPs with volume-loaded (i.e., 5.9 µL) and plate dimension (i.e., 10 × 13.503 cm) as the optimized solution, exhibiting desirability value of 1 and values of the CAAs, i.e., R_f_ of 0.65, peak height of 26876.2, capacity factor of 0.4127, theoretical plates of 41811.3 and separation number of 96.9, respectively (https://www.ncbi.nlm.nih.gov/pubmed/26912808).

**Supplementary Section 2:**

(2 A) Perfusate collection and analysis:

Steady-state during the SPIP studies was achieved within 30 min, after which aliquots of samples (1 mL each) were periodically withdrawn at regular time interval of 15 minutes each (i.e., 0, 15, 30, 45, 60). Meanwhile, the length and perimeter of the selected segments were determined. Diethyl ether (4 mL) was added to each perfusate sample (1 mL), and the mixture was centrifuged at 5,000 rpm (1,118 × g) for 20 minutes. Following centrifugation, 3 mL of the supernatant etheral fraction was collected. Finally, 1.5 mL of the perfusion solution was added to the ethereal extract and Mgf concentration in the mixture was determined spectrophotometrically at a λ_max_ of 254 nm. The effective permeability, wall permeability, fraction of dose absorbed and absorption number for all the treatment formulations were determined.

Perfusion data analysis

After achieving the steady state concentration, effective permeability (P_eff_) was calculated using Eq. (1) (Fagerho*lm et a*l., 1996; Mad*an et a*l., 2005):

$P*_{eff} = \left(1 - \frac{C_{m}}{C_{o}}\right) / 4 Gz$ **…Eq. (1)**

Where, C_m_ and C_o_ are the outlet and inlet concentrations of the perfusate solution concentration and Gz is Graetz number

Graetz number (Gz) is calculated from the following Eq. (2):

**Gz =$\left(\frac{\pi D L}{2 Q}\right)$ …Eq. (2)**

where, D, L, Q are the aqueous diffusion coefficient of the drug in perfusate, perfused intestinal length, and flow rate of perfusion, respectively.

Gz plays an important role in determining the dimensionless quantity, A.

| **0.004 ≤ Gz ≤ 0.01** | **A= 10.0 Gz + 1.01** |
| --- | --- |
| **0.01 ≤ Gz ≤ 0.03** | **A= 4.5 Gz+1.065** |
| **0.03 ≤ Gz** | **A = 2.5 Gz+ 1.125** |

Wall permeability was calculated using the Eq. (3):

$P*_{w} = \frac{P*_{eff} \times P*_{aq}}{P*_{aq} - P*_{eff}}$ **…Eq. (3)**

Dimensionless absorption number (A_n_) was computed using Eq. (4) as the ratio of fundamental mass transfer process affecting drug removal from intestine:

$A_{n} = SF \left(P*_{w} Gz\right)$ **…Eq. (4)**

where, SF is a scaling factor of 1.27 for complete radial mixing model.

The fraction of dose absorbed in rats was calculated using parameter determined from perfusion experiment using Eq. (5):

$F = 1 - e^{-2A_{n}}$ **…Eq. (5)**

Subsequently, the P_eff_ and F_a_ values were predicted in humans by interspecies scaling approach for the prepared formulations (Zakeri-Mila*ni et a*l., 2007). The conversion was performed using the Eq. (6) and (7), as follows:

$P_{eff}(human) = 11.04 P_{eff}(rat) - 0.0003$ **…Eq. (6)**

$F_{a}(human) = 1-e^{-38450} P_{eff} (rat)$ **…Eq. (7)**

**(2 B) Histology studies:** Histopathological evaluation of the vital organs of rats was conducted by excising all the organs, for all the groups were done by fixing a part in 10% w/v neutral buffered formalin solution for atleast 24 h. The process of fixation helps the tissue to prevent decomposition, putrefaction, and autolysis and withstand subsequent processing. Further, the tissue samples were processed by embedding these in paraffin wax, which were subsequently sectioned into slices of 5 μm thickness and stained with hematoxylin and eosin (H & E) for microscopic examination (Olympus, Tokyo, Japan).

Organs were blocked together, cut and serial sections were stained using hematoxylin and eosin (H&E); and Prussian blue and nuclear fast red respectively. Slides were visualized on a confocal microscope at 40X magnification. Eight images of non-overlapping areas of each organ of each mouse were taken for image quantification analysis after white-balancing the field of view for each slide.

**(2C) RBC Collection and morphology:**

The blood was collected after anesthesing the rats into 10 ml citrate-phosphate-dextrose anticoagulant vacuum tubes (Haematologic Technologies Inc., USA) by cardiac puncture. Harvested blood was centrifuged (2,200 × g, 10 min at 4 °C), and the plasma and buffy coat were removed by aspiration.

**Supplementary Section 3: Sample size was calculated using the formula**

Sample size=

Taking Type I error as 5%; , or was read from Z table, and the value was found to be 1.96.

And at 80% power, Type II error (β) terms out to be 100-80/100= 0.20; or was read from Z table, and the value was found to be 0.842.

The previous studies revealed that the standard deviation for AUC lies in between 25 to 35 h*ng.ml^-1^. Hence, the mean value was taken to be 30.

As the AUC for plain Mgf was 213 and for Mgf SPNMS was 8962, it indicated around 42 folds increase in AUC. Hence, the effect size for the (d), was found to be close to 42, Therefore, after substituting all the values in the above formula, sample size was calculated as follows:

Sample size ~ = 5.7 ~ 6

Thus, a total of 6 animals were taken for each time point for the pharmacokinetic studies. As there are a total of nine time-points and two withdrawals from one animal, 5x6 = 30 animals were employed in one group.

**Supplementary Section 4:**

Point-to-point level A linear correlations were attempted between the values of cumulative percent *in vivo* drug absorbed and the corresponding values of cumulative *in vitro* percent drug dissolved for Mgf SPNMS. The values of cumulative relative fraction absorbed at particular time-points were calculated employing modified Wagner-Nelson method.

Supplementary Figure 5 portrays statistically significant Level A correlation for individual animals in the Mgf SPNMS treated group. This corroborates that *in vitro* dissolution conditions, including drug release medium, apparatus, etc., are able to accurately predict the *in vivo* biopharmaceutical performance of the bioactive.


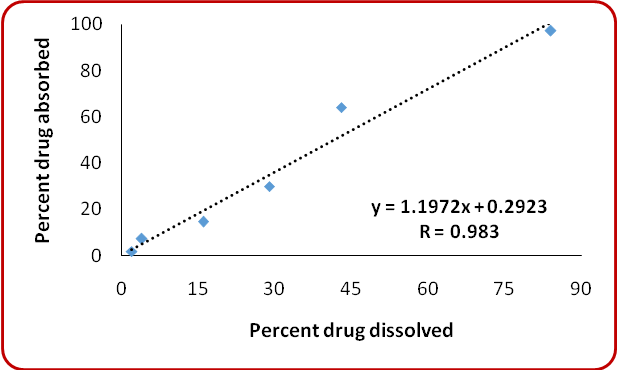


**Supplementary Figure 5:** Mean Level A correlation observed for animals (n=6) treated with Mgf SPNMS
